# Treatment of colorectal liver metastases

**DOI:** 10.1186/1477-7819-9-154

**Published:** 2011-11-24

**Authors:** Nabil Ismaili

**Affiliations:** 1 Department of medical oncology, Regional cancer center, Hassan II Hospital, Agadir-80000, Morocco

## Abstract

Colorectal cancer (CRC) is the third most common cancer in the word. Liver metastasis is the most common site of colorectal metastases. The prognosis of resectable colorectal liver metastases (CRLM) was improved in the recent years with the consideration of chemotherapy and surgical resection as part of the multidisciplinary management of the disease; the current 5-year survival rates after resection of liver metastases are 25% to 40%. Resectable synchronous or metachronous liver metastases should be treated with perioperative chemotherapy based on three months of FOLFOX4 (5-fluorouracil [5FU], folinic acid [LV], and oxaliplatin) chemotherapy before surgery and three months after surgery. In the case of primary surgery, pseudo-adjuvant chemotherapy for 6 months, based on 5FU/LV, FOLFOX4, XELOX (capecitabine and oxaliplatin) or FOLFIRI (5FU/LV and irinotecan), should be indicated. In potentially resectable disease, primary chemotherapy based on more intensive regimens such as FOLFIRINOX (5FU/LV, irinotecan and oxaliplatin) should be considered to enhance the chance of cure. The palliative chemotherapy based on FOLFIRI, or FOLFOX4/XELOX with or without targeted therapies, is the mainstay treatment of unresectable disease. This review would provide additional insight into the problem of optimal integration of chemotherapy and surgery in the management of CRLM.

## Background

Colorectal cancer (CRC) is the third most commonly diagnosed cancer in males and the second in females. It is the second most deathly cancer worldwide. About 1.2 million new cases and 608,700 deaths were reported to have occurred in 2008. However, the mortality rates have been decreasing dramatically in western countries largely resulting from improved treatment and increased awareness and early detection [1].

Liver is the most common site of metastasis from colorectal cancers (50-60% of the cases). Close to one third of patients have liver metastases either at the time of diagnosis (synchronous in 1/3 of the cases) or during the disease course (metachronous in 2/3 of the cases).

The prognosis of colorectal liver metastases (CRLM) has improved in the last few years. Surgical resection of liver metastases is considered the only curative treatment option for patients with resectable liver metastases and no extrahepatic disease [2,3]. Five years survival has increased from <8%, using palliative chemotherapy (CT), to 25-40% using multimodal management including CT and surgery [1,2,4-7]

Liver metastases are resectable in only 15% of the cases. Eighty five percents of the patients are ineligible to surgery because of the location, the size, the number of liver metastases, the residual normal liver, and the extra hepatic disease [2,8]. After primary surgery, the rate of relapse is high. This has led the investigators to evaluate the role of neoadjuvant and adjuvant CT in the management of these patients [9]. Furthermore, neoadjuvant CT is being increasingly used to downsize CRLM and render 10% to 30% of initially unresectable patients potentially resectable [8,10]. Local hepatic arterial infusion (HAI) CT after liver resection has proved to be effective; however, this technique is not widely used, because of concern of complications and the technical difficulties [11-13].

In general, the term neoadjuvant is used when the CT treatment is given preoperatively, adjuvant when the CT treatment is given postoperatively, and perioperative when the CT treatment is given both before and after surgery.

Limited data analyzed the role of targeted therapies (Bevacizumab and Cetuximab) in preoperative setting. The predictor factors such as K-RAS are crucial before the indication of optimal targeted therapy.

The present paper aimed to review the current data evaluating the role of preoperative, adjuvant, and perioperative CT in the management of CRLM.

### Strategies of literature research

The present review was based on a systematic literature search of Medline (Pubmed), last accessed 21 August 2011. The abstracts of papers presented at the annual meetings of the American Society of Medical Oncology (ASCO) were also analyzed. The key words used were: colorectal cancer, liver metastases, chemotherapy, targeted therapies, neoadjuvant, perioperative, adjuvant, steatohepatitis, and sinusoidal obstruction. All phases III and metanalyses are included. Selected phases II studies are also analyzed. Studies assessing local HAICT are excluded.

### Standard chemotherapy in metastatic setting

Prior to 2000, standard treatment for metastatic colorectal cancer was based on palliative CT using single-agent 5FU (or fluoropyrimidine drugs) combined with folinic acid (LV). The response rate with 5FU and folinic acid (5FU/LV) is approximately 20%. Initial randomized studies confirmed that regimen based on 5FU/LV, improved median survival of patients with metastatic disease from 8 to 12 months [14]. Subsequently the combination of 5FU with oxaliplatin typically FOLFOX or XELOX (capecitabine and oxaliplatin) and irinotecan typically FOLFIRI or XELIRI (capecitabine and irinotecan), has yielded overall response rate (ORR) of between 20-30% to 40-50%, and median overall survival (OS) of between 12 to 20 months [15-18].

The optimal sequencing of these two standard CT (FOLFOX or FOLFIRI) was evaluated by Tournigand and collaborators. They confirmed the survival equivalence of the two therapeutic sequences: FOLFOX first then FOLFIRI or FOLFIRI first then FOLFOX. However, the FOLFOX first was associated with a higher complete response rates (CRR) than FOLFIRI (4.5 vs. 2.8%). In addition, in the FOLFOX arm, 22% of patients received surgery for liver metastases vs. 9% in FOLFIRI arm (p = 0.02), leading to the usual use of FOLFOX CT in neoadjuvant setting. Toxicity profile of these 2 standard regimens was different. Grade 3/4 mucositis, nausea/vomiting, and grade 2 alopecia were more frequent with FOLFIRI, and grade 3/4 neutropenia and neurosensory toxicity were more frequent with FOLFOX [19].

A pooled analysis of seven randomized studies showed that survival is enhanced by the administration of all the three active agents, irrespective of their sequence. An interesting finding of this last analysis is that only 50-80% of patients are suitable for a second line CT and ultimately received the three drugs [20]. In the attempt to enhance treatment results and to increase the proportion of patients exposed to all active agents, a combined administration of 5FU/LV, irinotecan and oxaliplatin (FOLFIRINOX) has been developed. The FOLFIRINOX was evaluated in first line in comparison with the standard FOLFIRI, in 2 randomized phase III studies [21,22]. In a well designed study, FOLFIRINOX showed to be more effective (in ORR, progression free survival [PFS] and OS) than FOLFIRI and was associated with a higher secondary resection rate of liver metastases (36% vs. 12%; p = 0.017). This regimen was particularly toxic (grade 3/4 neutropenia = 50% vs. 28%) and requires special precautions [21]. The pooled analyses of theses 2 randomized studies confirmed these positives results [23]. FOLFIRINOX is an interesting regimen particularly in neoadjuvant setting for the management of potentially resectables CRLM.

The progress in molecular biology has prompted the investigators to develop new molecules targeting specific abnormalities in the cancer cells, with a very acceptable toxicity profile. Additional improvements in outcome have been associated with the use of biological agents in combination with cytotoxic CT. Two molecules are currently included in the first line treatment of metastatic CRC [24-29]. Bevacizumab is a humanized monoclonal antibody targeting the most important factor implicated in tumor angiogenesis called vascular endothelial growth factor (VEGF), and was the first molecule developed in the treatment of metastatic CRC. In first line, it was evaluated in randomized phase III trials in combination with IFL (5FU/LV bolus and irinotecan), FOLFIRI, FOLFOX, and XELOX. These studies confirmed the benefit of bavacizumab in ORR, PFS, and OS (table 1) [24-26]. Cetiximab is a chimeric monoclonal antibody targeting the epidermal growth factor receptor (EGFR). Two randomized trials in metastatic setting showed that adding cetuximab to FOLFIRI or FOLFOX, improved outcomes (ORR and PFS) in patients with K-RAS wild type tumors (table 2) [28,29]. With the use of bevacizumab and cetuximab in combination with CT, survival of patients has improved to more than 24 months.

**Table 1:** Randomized phase III trials evaluating Bevacizumab in combination with chemotherapy in first line treatment of metastatic CRC

Trials in first line treatment			Results		
**Trial name/Author**	**No**	**Treatments**	**ORR (%)**	**PFS (months)** **(first end point)**	**OS (months)**

Hurwitz (2004) [24]	813	IFL ± Bevacizumab	45 vs. 35 (p = 0.004)	10.6 vs. 6.2 (p < 0.001)	20.3 vs. 15.6 (p < 0.001)

BICC-C (2007) [25]	430	IFL/FOLFIRI ± Bevacizumab	57.9 vs. 53.3 (NS)	11.2 vs. 8.3 (p = 0.007)	NR vs. 19.2 (p = 0.007)

NO 16966 (2008) [26]	1401	XELOX/FOLFOX ± Bevacizumab	47 vs. 49 (NS)	9.4 vs. 8 (p = 0.0023)	21.3 vs. 19.9 (p = 0.077)

**Abbreviations**. ORR: overall response rate; DFS: disease free survival; OS: overall survival

**Table 2:** Randomized phase III trials evaluating Cetuximab in combination with chemotherapy in first line treatment of metastatic CRC

Trials in first line treatment (K-RAS wild type)			Results		
**Trial name/Author**	**No**	**Treatments**	**ORR (%)**	**PFS (months)** **(first end point)**	**OS (months)**

CRYSTAL (2009) [28] (phase 3)	599	FOLFIRI ± Cetiximab	59 vs. 43 (p = 0.03)	9.9 vs. 8.7 (HR = 0.68; 95% CI, 0.50 to 0.94)	23.5 vs. 20 (HR = 0.80)

OPUS (2009) [29] (phase 2)	338	FOLFOX ± Cetuximab	61 vs. 37 (p = 0.01)	8.3 vs. 7.2 (HR = 0.57; p = 0.016)	22.8 vs. 18.5 (HR = 0.86)

**Abbreviations**. ORR: overall response rate; PFS: progression free survival; OS: overall survival

### New staging system

A new staging system was proposed by the European Colorectal Metastases Treatment Group (ECMTG) that would subdivide the M from the TNM classification into 4 groups [8]:

*M0: no metastases

*M1a: resectable liver metastases

*M1b: potentially resectable liver metastases

*M1c: liver metastases those are unlikely to ever become resectable.

For both the M1a resectable patients and the M1b patients who become resectable after systemic treatment, resection offers the possibility of cure. For the M1c group, the possibility of doing a resection should not be excluded. Resection should be discussed in multidisciplinary team meetings.

### Criteria of resectability

There are at least three categories of patients with CRLM:

-First, the hepatic lesion(s) are clearly resectable at the time of presentation.

-Second, the hepatic lesion(s) are unresectable at presentation but potentially convertible to resection after primary CT called conversion CT.

-Third, the hepatic lesion(s) are unresectable and are unlikely to become resectable even with effective CT.

Relative contraindications of liver resection are synchronous rectal cancer; multiple, diffuse or large liver metastases; extrahepatic metastases; high level of ACE(> 200 ng/ml). In patients with no evidence of extrahepatic disease, the main contraindications to liver resection are tumors located close to the hepatic veins and inferior vena cava or to the liver hilum, larges, or numerous liver metastases. Contraindication usually results from a combination of these main causes that precludes liver resection with a sufficient margin of ≥ 1 cm. Curative resection is exceptional for patients with more than 4 metastases. These systems were established before the air of modern CT [6,30,31]

#### (A) French recommendations (FFCD)

The French recommendations divided the resectability on different classes [32,33]:

*Resectability of class I; easily resectable by experienced surgeons in liver surgery:

-Four or less than 4 segments involved and residual healthy liver volume > 40%:

-Vena cava free from tumor;

-≤1 hepatic vein,

-Contra-lateral portal pedicule.

*Resectability of class II; potentially resectable metastases by experienced surgeons in liver surgery: 5 to 6 segments involved ± contra-lateral major named vascular structures within liver. The hepatectomy is possible by a complex or large resection (more than 4 segments) that requires difficult and/or risky procedure (central hepatectomy, extended right hepatectomy, vascular reconstruction).

*Resection is not possible in the case of: (1) involvement of two portals branches; (2) involvement of one portal branch and a contra-lateral hepatic vein: or (3) involvement of three hepatic veins.

Prognostic factors are: Size > 5 cm, number > 4, bilobar character, invasion of pedicle lymph node, and/or high level of ACE.

#### (B) Oncosurge system

The oncosurge system define the respectability as the possibility of resection of all liver metastases with negative margins > 1 cm and a residual healthy liver volume > 20%. In this system, the prognostic factors are the performance status and the percentage of the underlying healthy liver. The extrahepatic metastases (hilar lymph nodes, lung, ovary, and/or adrenal metastases) are not a formal contraindication against surgery. Currentely approximately 20% of the patients with liver metastases can be resected, and have an estimated 5 years survival of 50% [34].

### Neoadjuvant chemotherapy

Neoadjuvant CT should be distinguished from conversion CT, which is also administered in the pre-operative setting but is administered to patients with initially unresectable disease, with the intention of downsizing the tumor burden, and, ultimately, considering resection.

#### (A) Conversion chemotherapy in initially unresectable colorectal liver metastases

Only a minority of patients with liver metastases is amenable directly to surgery (15%). Therefore, efforts have been made to increase the resectability of patients with initially unresectable colorectal liver metastases.

The downsizing of CRLM can have a number of advantages:

(1) small metastases can disappear in one lobe allowing resection of metastases in the opposite lobe;

(2) major vascular pedicles of the liver may become free from tumor;

(3) large lesions may become accessible to ablative techniques, when they shrink to less than 3 cm in diameter.

The role of conversion CT was evaluated in numerous retrospective and phase II studies [10,19,35-47]. In a large surgical series of 1104 patients with initially unresectable liver metastases, the 5 years survival of the resected patient (12%) following primary CT was 33%, that approached the 5 years survival rate of the resectable patients in the same period (equal to 48%) [10]. In a retrospective French study, 131 patients with unresectable liver metastases were included and received 3- 6 months of CT. In 57 patients (44%), curative surgery of liver metastases was considered possible. After 39 months median follow-up, the 4 years survival in resected patients was 37%. The rate of postoperative complication was 14% [35]. In addition, prospective studies confirmed the ability of neoadjuvant CT to render some metastases resectable. However, there is a wide differences in the liver resection rates reported for theses different trials reflecting in one part the differences in criteria for respectability/unresectability that exist between the different centers, in the absence of a clear definition. In another part, theses differences can be explained by the CT regimens used; in fact, FOLFOX, and FOLFIRINOX showed to be the most effective protocols (table 3) [19,36-47].

**Table 3:** Published prospective trials evaluating the resectability rate after first line-chemotherapy in patients with initially unresectable colorectal liver metastases

Auto (year)	No	Treatments	ORR	Resectability rate	Perioperative complications
Wein (2001) [36]	53	5FU/LV	41%	11%	Acceptable

Pozzo (2002) [37]	40	5FU/LV + irinotecan	47.5%	32.5%	Acceptable

Cals (2004) [38]	34	5FU/LV + irinotecan + oxaliplatin	50%	15%	Acceptable

Tourniguand (2004) [19]	109	FOLFOX	54%	22%	Acceptable
	
	111	FOLFIRI	56%	9%	Acceptable

Kohne (2005) [39]	216	5FU/LV + irinitecan	62.2%	7%	Acceptable
	
	214	5FU/LV	34.4%	3%	Acceptable

Ho (2005) [40]	40	5FU/LV/IRI	55%	10%	Acceptable

Seium (2005) [41]	30	5FU/LV + irinotecan + oxaliplatin	78%	23%	Hematological+++

Alberts (2005) [42]	42	FOLFOX	60%	40%	Acceptable

Masi (2006) [43]	74	FOLFIRINOX	72%	26%	

Ychou (2007) [44]	34	FOLFIRINOX	70.6%	26.5%	Acceptable Hematological+++

Falcone (2007) [21]	122	FOLFIRINOX	60%	15%	Hematological+++
	
	122	FOLFIRI	34%	6%	

Skof E (2009) [46]	41	XELIRI	49%	24%	Equivalent
	
	46	FOLFIRI	48%	24%	

Zhao R (2010) [47]	48	XELIRI	56.3%	42%	Diarrhea+++

**Abbreviations**. ORR: overall response rate

#### (B) Neoadjuvant chemotherapy in resectable colorectal liver metastases

Neoadjuvant CT is the administration of chemotherapy in the pre-operative setting in patients with resectable disease. It has a number of potential benefits:

(1) Increasing the percentage of resectability

(2) Achieving a limited hepatectomy

(3) Treatment of micro-metastases

(4) Evaluation of chemosensitivity of the disease and thus provides guidance about whether CT should be given after the resection of metastases.

The most important inconvenient of neoadjuvant CT is the progression of metastases during neoadjuvant CT.

**Figure 1:**
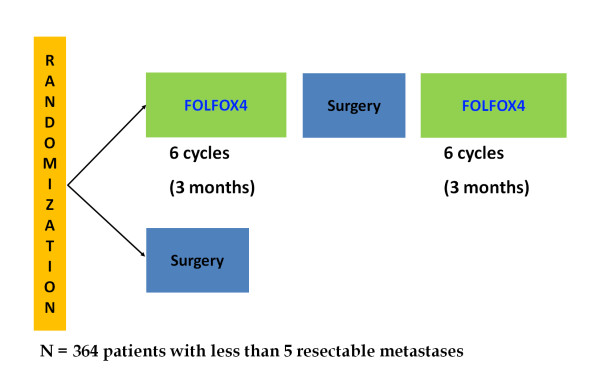
**The following figure shows the design of the EORTC Intergroup randomized trial 40983**.

The feasibility and the benefit of neoadjuvant CT have been evaluated in phase 2 studies and in one phase III randomized trial. In a phase 2 study (MIROX trial), the liver resection was performed after six cycles of FOLFOX CT. The ORR was 77% and the curative resection rate was 91%. The 2 year overall survival rate was 89%. The treatment was generally well tolerated; the main toxicities were grade 3 to 4 neutropenia and thrombocytopenia [48]. At present, only one randomized phase III trial investigating the role of perioperative CT in patients with resectable liver metastases has been published in the English literature. This parallel-group study conducted by the EORTC intergroup (EORTC Intergroup trial 40983) reports the final data for PFS. Three hundred 64 patients with colorectal cancer and up to 4 liver metastases were randomly assigned to either six cycles (3 months) of FOLFOX4 before and six cycles (3 months) after surgery or to surgery alone (182 in perioperative CT group vs. 182 in surgery group) (Figure 1). The primary objective was PFS. In the perioperative CT group, 151 (83%) patients were resected vs. 152 (84%) in the surgery group. The absolute increase in rate of PFS at 3 years was 7.3% (p = 0.058) in randomised patients; 8.1% (p = 0.041) in eligible patients; and 9·2% (p = 0.025) in patients undergoing resection. Reversible postoperative complications occurred more often in perioperative CT than group than in surgery alone group (25% *vs*. 16%; p = 0·04). Operative mortality was less than 1% in both treatment groups [9].

The combination of targeted agents with cytotoxic therapy showed high ORR and thus warrants assessment in the perioperative setting [24-29]. At least 4 phases II trials assessing the role of targeted therapies in preoperative setting were published (table 4) [49-52]. These studies showed that bevacizumab and cetuximab in combination with the standard CT improve ORR (70-78%) and resectability rates (60-93%) in patient with initially unresectable or potentially resectable CRLM, without inacceptable perioperative complications. Several randomized trials are ongoing to confirm theses preliminaries results. In a phase III study conducted by the EORTC group [EORTC 40051 BOS (Biologics, Oxaliplatin and Surgery) trial] the investigators assess the perioperative CT based on cetuximab plus FOLOFOX with or without bevacizumab in patients with resectable hepatic metastases from colorectal cancer [53]. In another phase III study, the NCI group investigates the benefit of adding cetuximab to FOLFOX in perioperative setting [54].

**Table 4:** Selected phase II trials investigating targeted therapies in preoperative setting

Auto (year)	Type of metastases	No	Treatments	Resectability rate	ORR	Perioperative complications
Gruenberger (2008) [49]	Potentially resectable	56	Bevacizumab + XELOX	93%	73% (CR = 8.9%; PR = 64.3%)	Acceptable

Wong (2011) [50]	Not suitable for upfront resection	46	Bevacizumab + XELOX	40%	78%	No grade 3-4 complications

Shimada (2011) [51]	Unresectable	7	Bevacizumab + FOLFIRINOX	71%	100%	No grade 3-4 complications

Folprecht (2010) [52]	Unresectable	114	Cetuximab + FOLFOX or FOLFIRI	60%	70% in K-RAS wild type tumors (R0 = 34%: 38% with FOLFOX vs 30% with FOLFIRI)	Acceptable

**Abbreviations**. ORR: overall response rate; CR: complete response; PR: partial response; R0: complete resection with free margins

### Adjuvant chemotherapy

In the past, the standard treatment of patients with resectable liver metastasis was surgical resection alone. Thereafter, CT (neoadjuvant or adjuvant) was introduced in the multidisciplinary management in light of several investigations.

Adjuvant systemic CT is defined by the administration of CT after complete resection of CRLM. It has the aim to reduce the risk of recurrence and to improve patient survival.

There are 2 theoretical rational for adjuvant CT after liver resection: (1) the presence of cancer cells "dormant" in the remaining liver; and (2) the benefit of adjuvant CT after surgery for stage III colorectal cancers [55].

In analogy to stage III disease, adjuvant CT or pseudo-adjuvant CT was assessed after the complete resection of all liver disease in two phase III trials using a similar design, but closed prematurely because of slow accrual; The pooled analysis based on individual data from these two trials was also published. A third and fourth phase III studies were conducted to compares two different adjuvant CT regimens (table 5) [56-60].

**Table 5:** Randomized trials and metaanalyses evaluating the role systemic adjuvant chemotherapy in patients with completely resected colorectal liver metastases

Authors (year)	Type of study	No	Randomized postoperative chemotherapy	Median PFS (months)	Median OS (months)
Langer B (2002) [56]	Phase III	129	5FU/LV	No difference	No difference

Portier (2006) [57]	Phase III	173	5FU/LV	24.4 vs. 16.6 (p = 0.028)	62.1 vs. 46.4 (p = 0.13)

Mirty (2006) [58]	Pooled analysis	278	5FU/LV	27.9 vs. 18..8 (p = 0.059)	61.1 vs. 46.9 (p = 0.125)

Ychou (2009) [59]	Phase III	306	FOLFIRI vs. 5FU/LV	24.7 vs. 21.6 (p = 0.44)	No difference

Voest (2011) [60]	Phase III	79	Bevacizumab + XELOX vs. XELOX	2 years PFS = 70% vs. 52% (p = 0.074)	-

Abbreviations. PFS: progression free survival; OS: overall survival

In the first 2 phase III studies, the patients were randomized between two arms, one experimental arm testing adjuvant CT containing 5FU/LV bolus regimen for 6 months, and a control arm treated by surgery alone. In the first study (Fédération Francophone de Cancérologie Digestive [FFCD] Trial 9002)including 173 patients, the differences in 5-year PFS and 5-year OS between the two arms were not significant, 33.5% vs. 26.7% and 51% vs. 42%, respectively. However, it has shown a PFS benefit for CT in multivariate analysis (Cox model for PFS: odds ratio = 0.66, P = 0.028) [57]. The second randomized study is a multicenter study conducted by the EORTC/NCIC/GIVIO group, (European Organization for Research and Treatment of Cancer/National Cancer Institute of Canada Clinical Trials Group/Gruppo Italiano di Valutazione Interventi in Oncologia [ENG] trial), including 129 patients. The results at 4 years did not show any difference between the 2 groups either in PFS (45% vs. 35%) and OS (57% vs. 47%) [56]. The pooled analysis using the individual data from these two studies was presented at the ASCO 2006 annual meeting and was recently published. This meta-analysis showed a marginal statistical significance in PFS (p = 0.095) and OS (p = 0.058) in favor of adjuvant chemotherapy. In addition, a multivariate analysis of three factors (treatment groups, the number of metastases, and relapse-free interval before metastasis), suggested the benefit for CT [58].

A third randomized study comparing two adjuvant CT treatments, FOLFIRI and 5FU/LV, was conducted and recently published by a French team. Three hundred 22 completely resected (R0) patients were included in this study. In the published article, the authors concluded that there were no differences in PFS (first end point) and in OS between the 2 CT arms. However, in the patients treated early within 42 days of surgery, the FOLFIRI CT same to do better than 5FU/LV CT (PFS: p = 0.17) [59].

Finally, a fourth trial has compared adjuvant treatment associating bevacizumab to XELOX vs. XELOX alone after radical resection of CRLM. Due to slow accrual, the study was closed early, after inclusion of 79 patients. At last follow-up, the 2 year PFS survival rate (first end point) was 70% vs. 52% in favor of Bevacizumab arm, however, the difference was not significant (p = 0.074). No significant differences in toxicity between the 2 arms were found [60].

### Liver injuries related to neoadjuvant chemotherapy

Although preoperative CT has many advantages, there has been growing concern about the potential for hepatotoxicity. The types of injuries observed in the liver specimens from patients treated with preoperative CT include steatosis, steatohepatitis, and sinusoidal injuries. These pathologies have showed to be drug specific as well as related to the duration of CT.

#### (A) Steatosis and steatohepatitis

Steatosis is defined by the accumulation of lipids in the hepatocyte. Its prevalence rages between 16 to 31% increasing to 46-75% in heavily consumed alcohol and obese patients. In the later stages, steatosis is accompanied by inflammation and balloonisation which lead to fibrosis, and is termed steatohepatitis. Steatohepatitis can lead to significant decrease in liver function. Over a 10-year period, approximately 9%-20% of patients with steatohepatitis develop cirrhosis. Of the patients who develop cirrhosis, 22%-33% of them develop end-stage liver disease [61-66]. Analysis of the impact of steatosis on outcome after liver resection suggests that morbidity is increased but not mortality [67,68]. While steatohepatitis may be associated with increased 90-day mortality due to liver failure after surgery [69].

#### (B) Vascular damage or sinusoidal obstruction syndrome

Sinusoidal obstruction syndrome results from damage to endothelial cells lining the sinusoids of the liver [70,71]. It can lead to portal hypertension, ascites, hyperbilirubinemia, and in severe cases, liver failure. One of the sign of sinusoidal obstruction syndrome is sinusoidal dilation. Analysis of the impact of vascular lesions on outcome following liver resection suggests that sinusoidal injury increases the risk of operative bleeding but does not increase perioperative morbidity and mortality [69,72].

#### (C) Drug specific toxicity

Liver damage following CT has showed to be drug specific as well as related to the duration of CT. It was reported that 5FU can be associated with an increased risks of severe steatosis [73]. Oxaliplatin based combination regimen is associated with an increased risk of vascular lesions of the liver [69,72,74]. In other reports, irinotecan-containing regimens was associated with increased risks of steatosis and steatohepatitis [67,69,75]

VEGF plays a critical role in liver regeneration. Consequently, it is possible that hepatic regeneration could be reduced in patients undergoing liver resection following VEGF blockage [76]. The results of 2 preclinical investigations are inconsistent regarding the effect of EGFR inhibition on hepatic regeneration; the first study showed strong evidence that EGFR is essential in hepatic regeneration, however, the second study showed that cetuximab does not adversely affect liver resection in mice [77,78]. Until further evidence is obtained, it is reasonable to allow a 6/8 week interval between the last administration of bevacizumab/cetuximab and surgery.

#### (D) The impact of chemotherapy duration

Two studies clearly showed that the morbidity rate is related to the duration of CT administered. In the first study with more than 12 courses of CT, was associated with higher risk of post operative complications compared with < or 12 courses. In the second study, the authors showed that postoperative morbidity was higher in patients receiving more than 6 cycles of CT before surgery [72,79]. More recently, safety data of the EORTC 40983/EPOC phase III study showed that the administration of 6 cycles of FOLFOX before surgery appears feasible; the mortality rate was very low (close to 1%) and the rate of reversible complications was acceptable [9].

### Treatment recommendations (figure 2) [32,33,80]

**Figure 2:**
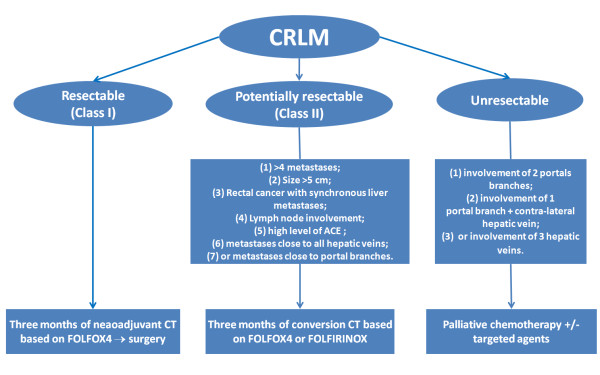
**The following figure summarizes the treatment recommendations**.

Treatment choices for patients with CRLM should be discussed in multidisciplinary team meetings (surgeon, medical oncologist, radiation oncologist, radiologist, pathologist...)

#### (A) Resectable colorectal liver metastases

Perioperative treatment with 3 months (6 cycles) of FOLFOX4 CT was compatible with major liver surgery, improved PFS in resectable patients, and should be considered as a standard of care in patients with resectable CRLM.

In the case of primary surgery, adjuvant CT based on 5FU/LV, FOLFOX4/XELOX or FOLFIRI regimens for 6 months should be considered as an option in patient with completely resected liver metastases.

#### (B)Potentially resectable colorectal liver metastases

Neoadjuvant CT called conversion CT based on 3 months (6 cycles) of FOLFOX4 or FOLFIRINOX regimens should be considered to enhance the chance of cure of patient with initially unresectable liver metastases. Resection should be discussed in multidisciplinary team meetings.

#### (C)Liver metastases those are unlikely to ever become resectable

Palliative CT based on FOLFOX4/XELOX, FOLFIRI, with or without biological therapies should be considered.

In this setting, the possibility of doing a resection should not be excluded. Resection should be discussed in multidisciplinary team meetings.

Table 6 summarizes the most used CT regimens in metastatic CRC.

**Table 6:** The most used chemotherapy regimens in the treatment of metastatic colorectal cancer

Regimen	Cycle length	Drogues and doses				
	
		1	2	3	4	5
5FU Mayo clinic	4 weeks	LV 20 mg/m²/D on D 1-5	5FU 425 mg/m²/D bolus on D 1-5	-	-	-

5FU/LV (de Gramont) [17]	2 weeks	LV 200 mg/m² over 2 h on D1 and D2	5FU 400 mg/m² bolus on D1 and D2	5FU 600 mg/m² CIVI over 22 h on D1 and D2	-	-

FOLFOX4 [17]	2 weeks	Oxaliplatin 85 mg/m² over 2 h on D1	LV 200 mg/m² over 2 h on D1 and D2	5FU 400 mg/m² bolus on D1 and D2	5FU 600 mg/m² CIVI over 22 h on D1 and D2	-

Modified FOLFOX6	2 weeks	Oxaliplatin 85 mg/m² over 2 h on D1	LV 200 mg/m² over 2 h on D1 and D2	5FU 400 mg/m² bolus on D1	5FU 2400 mg/m² CIVI over 46 h on D1	

XELOX	3 weeks	Oxaliplatin 130 mg/m² over 2 h on D1	Capecitabine 1 g/m² BD on D1-14	-	-	-

FOLFIRI [19]	2 weeks	Irinotecan 180 mg/m² over 1 h on D1	LV 200 mg/m² over 2 h on D1 and D2	5FU 400 mg/m² bolus on D1 and D2	5FU 600 mg/m2 CIVI over 22 h on D1 and D2	-

XELIRI	3 weeks	Oxaliplatin 130 mg/m² over 2 h on D1	Capecitabine 1 g/m² BD on D1-14	-	-	-

FOLFIRINOX (Italy) [21]	2 weeks	Irinotecan 165 mg/m² over 1 h on D1	Oxaliplatin 85 mg/m² over 2 h on D1	LV 200 mg/m² on D1	5FU 400 mg/m² bolus on D1	5FU 3200 mg/m2 CIVI 48 h on D1

FOLFIRINOX (France) [45]	2 weeks	Irinotecan 180 mg/m² over 1 h on D1	Oxaliplatin 85 mg/m² over 2 h on D1	LV 400 mg/m² on D1	5FU 400 mg/m² bolus on D1	5FU 2400 mg/m2 CIVI 48 h on D1

Bevacizumab + FOLFOX4 [26]	2 weeks	Bevacizumab 5 mg/kg over 30-90 min on D1	Oxaliplatin 85 mg/m2 over 2 h D1	LV 200 mg/m² over 2h on D1 and D2	5FU 400 mg/m² bolus on D1 and D2	5FU 600 mg/m² CIVI over 22 h on D1 and D2

Bevacizumab + XELOX [26]	3 weeks	Bevacizumab 7.5 mg/kg over 30-90 min on D1	Oxaliplatin 130 mg/m² over 2 h on D1	Capecitabine 1 g/m² BD on D1-14	-	-

Bevacizumab + FOLFIRI [25]	2 weeks	Bevacizumab 5 mg/kg over 30-90 min on D1	Irinotecan 180 mg/m² over 1 h D1	LV 200 mg/m² over 2h on D1 and D2	5FU 400 mg/m² bolus on D1 and D2	5FU 600 mg/m² CIVI over 22 h on D1 and D2

Cetuximab + FOLFIRI (K-RAS wild type) [28]	2 weeks	Cetuximab 400 mg/m² over 2 h on D1 then 250 mg/m² weekly	Irinotecan 180 mg/m² over 1 h on D1	LV 200 mg/m² over 2 h on D1 and D2	5FU 400 mg/m² bolus on D1 and D2	5FU 600 mg/m² CIVI over 22 h on D1 and D2

Cetuximab + FOLFOX (K-RAS wild type) [29]	2 weeks	Cetuximab 400 mg/m² over 1 h on D1 then 250 mg/m² weekly	Oxaliplatin 85 mg/m² over 2 h on D1	LV 200 mg/m² over 2h on D1 and D2	5FU 400 mg/m² bolus on D1 and D2	5FU 600 mg/m2 CIVI over 22 h D1 and D2

Abbreviations: D = day; CIVI = continuous intravenous infusion; BD = bid day

## Conclusion

**Figure 3:**
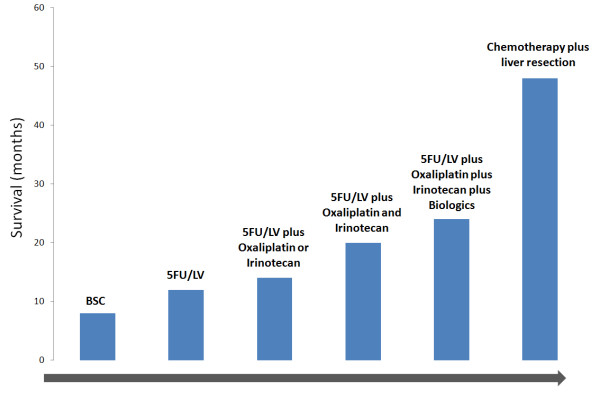
**Figure illustrates the improvement in survival of patients with CRLM by the development of modern chemotherapy and targeted therapies and mainly by the consideration of resection as part of the multidisciplinary management**.

The prognosis of patients with metastatic colorectal cancer of the liver has improved significantly over the last few years (Figure 3). The management has become multidisciplinary using modern CT regimens and more developed surgical techniques. For patients with resectable metastases, perioperative CT based on FOLFOX4 is the treatment of choice before surgical resection according to the results of the EORTC40983 randomized trial. For patient with resectable metastases treated with primary surgery, adjuvant CT is a reasonable option. In the case of initially unresectable disease, conversion CT using more active combination such as FOLFIRINOX should be indicated to downsizing liver metastases and to optimize the chance of cure. If metastases are unresectable and unlikely to be resectable, palliative CT with or without targeted agents is the mainstay treatment. The role of targeted therapy in neoadjuvant setting will be defined in the near future. Therapeutic investigations should be continued with the development of more efficient regimens, newer surgical and ablative techniques, to improve treatment results of colorectal liver metastases.

## Abbreviations

**CRC **: colorectal cancer; **CRLM **: colorectal liver metastases; **CRR **: complete response rate; **CT **: chemotherapy; **EGFR **: epidermal growth factor receptor; **5FU : **5-fluorouracil; **HAI **: **hepatic arterial infusion; LV **: folinic acid; **ORR **: overall response rate; **OS **: overall survival; **PFS **: progression free survival (or disease free survival); **VEGF **: vascular endothelial growth factor.

## Competing interests

The author declares that they have no competing interests.

## Authors' contributions

NI is involved in concept design, in data collection, drafting and critically revising the manuscript.
